# Thoughts from SNP-SIG 2012: future challenges in the annotation of genetic variations

**DOI:** 10.1186/1471-2164-14-S3-S1

**Published:** 2013-05-28

**Authors:** Yana Bromberg, Emidio Capriotti

**Affiliations:** 1 Department of Biochemistry and Microbiology, Rutgers University, New Brunswick, NJ, USA; 2 Division of Informatics, Department of Pathology, University of Alabama at Birmingham, Birmingham, AL, USA

## Overview

Advances in high-throughput sequencing, genotyping, and characterization of haplotype diversity are consistently generating vast amounts of genomic data. Single Nucleotide Polymorphisms (SNPs) are the most common type of genetic variation [1]. In the recent years the number of known SNPs has been increasing exponentially [2]; the last release of the NCBI's dbSNP database [3] contained more than 55 million human SNPs. SNPs are interesting as both markers of evolutionary history and in the context of their phenotypic manifestations (e.g. characteristic traits and diseases). However, due to their sheer number, detailed experimental annotations are impossible and computational inference is severely limited by the required resources. SNPs also present a challenge for visualization and storage.

In line with the increasing interest in the genetic variation analysis and annotation, on July 14th, 2012 [4] we organized the second SNP Special Interesting Group (SNP-SIG) meeting at the ISMB'12 in Long Beach, CA. This meeting attempted to summarize the field's research advances in the directions of "Annotation and prediction of structural/functional impacts of coding SNPs" and "SNPs and Personal Genomics: GWAS, populations and phylogenetic analysis". The discrepancy between the significant availability of the SNP data and the current lack of its interpretation requires the development of new computational annotation methods. The analysis of genetic variation is a key factor for the understanding of the genomic information. The SNP-SIG provides a forum necessary for the organization of a research network facilitating the exchange of ideas and for the establishment of new collaborations to manage the complexity of the analysis of genetic variation. The one-day SIG attracted over 80 participants, with nine "bleeding-edge" research talks and five presentations from the leading scientists in the field.

The topics covered in the SIG presentations focused primarily on annotating the phenotypic traits associated with the specific SNPs - everything from annotating associated protein function changes [5-10] to screening pharmocogenomic variants [11] to finding driver mutations in cancer [12,13]. There was also some focus on improving method performance, *e.g*. a new way to efficiently analyze multi-GWAS data [14]. Finally, the issues of the quality of the current experimental variant annotations were also discussed. While the overall focus of the SIG was clearly on the side of improving variant annotation accuracy, the participants clearly recognized the need for faster methods capable of dealing with larger sets and noisy data. Although recognized as existing issues, the visualization of the SNP data and increasing data storage concerns, were not explicitly addressed. These will be a stressed focus in the future SNP-SIG meetings.

## Next meeting

We are currently preparing for the next edition of the SNP-SIG meeting to be held in the context of the ISMB 2013, Berlin, Germany. Further information about the SNP-SIG 2013 is available on our web site (http://snpsig.biofold.org).
